# Skin Cancer Prevention Behaviors Among Agricultural and Construction Workers in the United States, 2015

**DOI:** 10.5888/pcd16.180446

**Published:** 2019-02-07

**Authors:** Kathleen R. Ragan, Natasha Buchanan Lunsford, Cheryll C. Thomas, Eric W. Tai, Aaron Sussell, Dawn M. Holman

**Affiliations:** 1 Centers for Disease Control and Prevention, National Center for Chronic Disease Prevention and Health Promotion, Division of Cancer Prevention and Control, Atlanta, Georgia; 2 Centers for Disease Control and Prevention, National Institute for Occupational Safety and Health, Spokane Mining Research Division, Spokane, Washington

## Abstract

**Introduction:**

Nearly 5 million people are treated for skin cancer each year in the United States. Agricultural and construction workers (ACWs) may be at increased risk for skin cancer because of high levels of ultraviolet radiation exposure from the sun. This is the first study that uses nationally representative data to assess sun-protection behaviors among ACWs.

**Methods:**

We analyzed data from the 2015 National Health Interview Survey Cancer Control Supplement to examine the prevalence of sun-protection behaviors among ACWs. We calculated national, weighted, self-reported prevalence estimates. We used χ^2^ tests to assess differences between ACWs by industry and occupation.

**Results:**

Most of the 2,298 agricultural and construction workers studied were male (by industry, 72.4% in agriculture and 89.3% in construction; by occupation, 66.1% in agriculture and 95.6% in construction) and non-Hispanic white. About one-third had at least 1 sunburn in the past year. The prevalence of sunscreen use and shade seeking was low and did not significantly differ among groups, ranging from 15.1% to 21.4% for sunscreen use and 24.5% to 29.1% for shade seeking. The prevalence of wearing protective clothing was significantly higher among agricultural workers than among construction workers by industry (70.9% vs 50.7%) and occupation (70.5% vs 53.0%).

**Conclusion:**

Our findings could be used to improve occupational health approaches to reducing skin cancer risk among ACWs and to inform education and prevention initiatives addressing skin cancer. Sun-safety initiatives may include modifying work sites to increase shade and adding sun safety to workplace policies and training. Employers can help reduce occupational health inequities and protect workers by creating workplaces that facilitate sun protection.

**Summary**

**What is already known on this topic?**

Agricultural and construction workers (ACWs) may be at increased risk for skin cancer because of high levels of ultraviolet radiation exposure from the sun.

**What is added by this report?**

Agricultural workers had a higher prevalence than construction workers of almost all sun-protection behaviors. Prevalence of regular use of shade and sunscreen was lower among ACWs than national estimates.

**What are the implications for public health practice?**

Findings may be used to improve occupational health approaches to skin cancer risk reduction among ACWs. Employers can help reduce occupational health inequities and protect workers by creating workplaces that promote sun-safe policies, provide access to resources that facilitate sun protection, and foster workplace cultural sun-safety expectations.

## Introduction

Nearly 5 million people are treated for skin cancer annually in the United States (1). Incidence rates are highest among men and non-Hispanic white people. Overexposure to ultraviolet (UV) radiation is the primary cause of most skin cancers. Agricultural and construction workers (ACWs), who spend most of their work-related time outdoors, may be at increased risk for skin cancer because of high levels of UV radiation exposure from the sun (2). Although data on skin cancer–associated risk among such workers are limited, studies assessing occupational associations with other health risk behaviors (eg, smoking) and chronic diseases (eg, obesity) have been explored (3–7). Compared with workers in other occupations, ACWs have poorer physical and mental health outcomes (8–12).

In 2014, the US Surgeon General described prevention strategies in the *Call to Action to Prevent Skin Cancer,* highlighting the need for more research on sun-protection strategies among outdoor workers (13). The objective of our study was to examine the prevalence of skin cancer prevention behaviors among ACWs. Ours is the first known US study to assess skin cancer prevention behaviors among these workers by using nationally representative data. Findings can help inform opportunities for improvements in health education and cancer prevention initiatives for ACWs, community members, and employers of outdoor workers.

## Methods

The National Health Interview Survey (NHIS) is an annually administered, nationally representative, cross-sectional household, in-person survey of the US noninstitutionalized civilian population. A core set of demographic and health data are collected annually along with supplemental data. Details on survey methods are available elsewhere (14). We used data from the 2015 Cancer Control Supplement included in the Sample Adult section. Because we used existing publicly available de-identified data, our study was exempt from review by the Centers for Disease Control and Prevention (CDC) Human Subjects Institutional Review Board. Analyses were performed from February to June 2018.

The Sample Adult section had a response rate of 55.2% (14). Respondents aged 18 or older were asked about their employment status during the week before their interview. Industry and occupation information was recorded verbatim from respondents who reported they 1) were working for pay at a job or business, 2) had a job or business but were not at work, 3) were working, but not for pay, at a family-owned job or business, or 4) were not currently working but had previously worked. Industry refers to employer type of business or work (eg, dairy farm), whereas occupation refers to type of work (eg, farm hand). US Census Bureau coding specialists reviewed responses and assigned 4-digit census codes based on the 2012 North American Industry Classification System and the 2010 Standard Occupational Classification (14). To prevent inferential disclosure of identity in the NHIS public data sets, CDC recodes the industry and occupation census codes into less specific groups: NHIS 2-digit “detailed” and “simple” recodes. We used the simple recodes to obtain reliable estimates, given the relatively small sample sizes for agricultural and construction industry and occupation groups.

Our sample consisted of respondents who reported that their main, current, or most recent job was as an agricultural worker or construction worker (n = 2,747). Agricultural workers were defined as respondents with an NHIS industry recode of 01 (denoting “Agriculture, Forestry, Fishing, and Hunting Industries”) or an NHIS occupation recode of 18 (denoting “Farming, Fishing, and Forestry Occupations”). Construction workers were defined as respondents with an NHIS industry recode of 04 (denoting “Construction Industries”) or an NHIS occupation recode of 19 (denoting “Construction and Extraction Occupations”). Because extraction occupations are primarily in mining industries, workers with an NHIS industry recode of 02 (denoting “Mining Industries” [n = 112]) were excluded from the “Construction and Extraction Occupations” group to further define the sample group of “Construction Occupations.” Respondents who had a family history of melanoma (n = 38) or had a skin cancer diagnosis (including melanoma, nonmelanoma, other/unknown [n = 102]) were excluded from analyses because their awareness of skin cancer risk and sun-protection behaviors may be higher than that among respondents without a family or personal skin cancer history, and thus, they may not be representative of the group of workers without a skin cancer history. Some respondents (n = 218) had a response of “do not go out in the sun” for 1 or more of the sun sensitivity or sun-protection questions. Because this response was coded according to a respondent’s statement and was not explicitly provided by the interviewer, we excluded respondents with this response. The total number of workers excluded from the industry and occupation groups of interest was 449 of 2,747 (16.3%) (Figure), thus yielding a final sample size of 2,298 ACWs.

**Figure:**
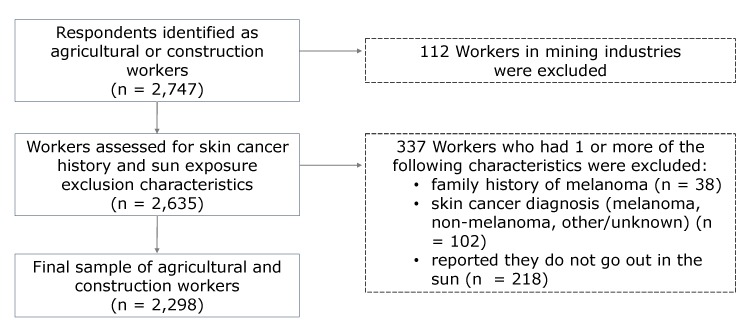
Industry and occupation data from the 2015 National Health Interview Survey were used to identify 2,747 agricultural and construction workers. A total of 449 workers were excluded from our study on sun-protection behaviors, yielding a final sample of 2,298 agricultural and construction workers.

Our main outcomes of interest were 5 sun-protection behaviors (staying in the shade, wearing a wide-brimmed hat, wearing a long-sleeved shirt, wearing long pants or other clothing that reaches the ankles, and using sunscreen with a sun protection factor [SPF] ≥15) (15). Sun-protection behaviors were measured by using a 5-point Likert scale (always, most of the time, sometimes, rarely, never) with the prompt, “When you go outside on a warm sunny day for more than one hour, how often do you . . . ?” We classified responses into 3 levels: 1) always/most of the time, 2) sometimes/rarely, and 3) never. We included an item on using a baseball cap or sun visor so that we could compare the use of these with the use of a wide-brimmed hat. Participants who reported using sunscreen were asked about the SPF of the sunscreen used most often. The minimum recommendation for protection against skin cancer and sunburn is SPF 15 (16); therefore, we excluded participants who indicated using an SPF <15 from analyses on sunscreen use. We created dichotomous variables to capture data on respondents who used any method of sun protection and respondents who used at least 1 form of protective clothing always or most of the time. We calculated a continuous overall sun-protection behavior score (ranging from 0 to 4, with 4 indicating always doing the behavior and 0 indicating never for all behaviors) by using the average of scores from the 5 sun-protection behaviors.

Demographic characteristics were sex, age, race/ethnicity, educational attainment, marital status, nativity (born in the United States or not), US region, health insurance coverage, and paid sick leave. Respondents’ skin sensitivity to short-term (1 hour) and repeated (every day for 2 weeks) sun exposure while unprotected (without sunscreen, a hat, or protective clothing) was measured. We assessed sunburn history (during the past 12 months) by recoding the continuous measure into an ordinal variable (0, 1, ≥2 sunburns).

To account for the complex survey design, we analyzed data by using SAS-callable SUDAAN, version 11.0 (RTI International). We calculated descriptive statistics and national, weighted, self-reported prevalence estimates (with 95% confidence intervals [CIs]) by the NHIS simple recode categories of agricultural and construction industries and occupations. Estimates with a sample size of less than 30 were not tabulated, and estimates with a sample size of 30 to 59 were flagged and should be interpreted with caution (17). We used χ^2^ tests to assess differences between ACWs by industry and occupation, and we used a significance level of *P* < .05.

## Results

By industry group, 468 respondents were in agriculture and 1,630 were in construction. By occupation, 261 respondents were in agriculture, and 1,313 were in construction (Table 1). Most respondents were male; this percentage ranged from 66.1% to 72.4% in agriculture and 89.3% to 95.6% in construction. Most were non-Hispanic white; this percentage ranged from 44.4% to 60.2% in agriculture and 60.2% to 64.7% in construction. About half of workers in agricultural occupations were Hispanic (50.5%) and had less than a high school education (49.2%). A greater percentage of construction workers than agricultural workers had at least some college. Most ACWs were married or living with a partner and born in the United States. Most who were not US-born reported living in the United States for 15 years or more.

**Table 1:** Weighted Percentages (95% CI) of Demographic Characteristics of Agricultural and Construction Workers (N = 2,298), by Industry and Occupation,^a^ National Health Interview Survey, United States, 2015^b^

Variable	Industry		Occupation	
	Agriculture, Forestry, Fishing, and Hunting (n = 468) (Weighted n = 2,595,261)	Construction (n = 1,630) (Weighted n = 12,028,139)	Farming, Fishing, and Forestry (n = 261) (Weighted n = 1,487,772)	Construction (n = 1,313) (Weighted n = 8,974,654)
**Sex**				
Male	72.4 (66.0–77.9)	89.3 (87.0–91.3)	66.1 (56.2–74.8)	95.6 (94.1–96.8)
Female	27.6 (22.1–34.0)	10.7 (8.7–13.0)	33.9 (25.2–43.8)	4.4 (3.2–5.9)
**Age, y**				
18–29	17.7 (13.7–22.6)	15.4 (13.1–18.1)	6.0 (18.9–34.8)^c^	17.3 (14.4–20.7)
30–39	16.8 (12.8–21.8)	23.1 (20.4–26.1)	17.6 (11.7–25.6)^c^	22.2 (19.3–25.3)
40–49	18.3 (13.6–24.3)	18.5 (15.9–21.3)	19.9 (13.9–27.6)^c^	19.1 (16.3–22.3)
50–65	27.7 (23.2–32.8)	29.3 (26.4–32.3)	24.6 (19.6–30.5)	27.4 (24.3–30.7)
≥66	19.5 (14.9–25.0)	13.7 (11.7–16.1)	11.9 (7.9–17.6)^c^	14.0 (11.9–16.5)
**Race/ethnicity**				
Non-Hispanic white	60.2 (52.4–67.5)	64.7 (61.4–67.9)	44.4 (33.5–55.8)	60.2 (56.5–63.8)
Non-Hispanic black	^—c^	6.6 (5.3–8.2)	^—c^	7.2 (5.7–9.0)
Non-Hispanic other	^—c^	2.9 (2.1–4.0)	^—c^	2.9 (2.0–4.2)^c^
Hispanic	33.1 (25.4–41.7)	25.8 (23.1–28.8)	50.5 (39.1–61.7)	29.7 (26.5–33.2)
**Educational attainment**				
Less than high school	35.4 (28.8–42.7)	23.4 (20.7–26.3)	49.2 (37.8–60.7)	29.2 (25.7–32.9)
High school graduate/GED	32.6 (26.4–39.5)	34.4 (31.3–37.6)	27.8 (19.8–37.5)	35.6 (32.0–39.4)
Some college, no degree	14.5 (10.3–20.1)	18.1 (15.8–20.7)	12.8 (8.1–19.8)^c^	17.3 (14.7–20.3)
Associate degree^d^	5.0 (2.8–8.7)^c^	13.3 (10.7–16.2)	^—c^	12.6 (10.3–15.4)
Bachelor’s degree or higher	12.5 (8.6–17.7)	10.9 (8.9–13.3)	^—c^	5.2 (3.8–7.1)
**Marital status**				
Married or living with partner	65.8 (59.3–71.8)	70.4 (67.2–73.4)	59.9 (50.2–68.9)	66.2 (62.4–69.9)
Divorced, separated, or widowed	15.7 (12.2–20.0)	11.5 (9.9–13.2)	14.6 (10.0–20.8)^c^	12.9 (10.9–15.2)
Never married	18.5 (13.8–24.4)	18.2 (15.7–20.9)	25.5 (18.0–34.9)	20.9 (18.0–24.1)
**Nativity**				
Born in the United States	71.1 (61.5–79.1)	77.4 (74.5–80.1)	57.8 (44.7–70.0)	75.4 (71.9–78.6)
<15 y in the United States	9.2 (5.8–14.5)^c^	7.3 (5.8–9.2)	14.3 (9.7–20.8)^c^	8.3 (6.5–10.5)
≥15 y in the United States	19.7 (14.1–26.8)	15.3 (13.2–17.6)	27.8 (19.4–38.3)	16.4 (13.7–19.4)
**US region** e				
Northeast	9.8 (5.8–16.2)^c^	16.9 (14.5–19.7)	^—c^	15.0 (12.6–17.7)
Midwest	27.1 (21.1–34.2)	21.3 (18.5–24.5)	18.2 (11.6–27.2)^c^	21.9 (18.6–25.5)
South	24.0 (18.6–30.5)	37.4 (34.4–40.6)	21.7 (15.4–29.6)^c^	38.0 (34.5–41.6)
West	39.0 (31.7–46.8)	24.3 (21.8–27.1)	51.6 (41.4–61.7)	25.2 (22.2–28.4)
**Health insurance coverage**				
Private	48.8 (41.8–55.9)	54.4 (50.9–57.8)	32.4 (24.3–41.8)	49.0 (45.2–52.7)
Public^f^	30.7 (25.4–36.5)	23.9 (21.2–26.9)	44.6 (36.8–52.7)	24.5 (21.5–27.7)
None	20.5 (15.1–27.2)	21.7 (19.1–24.6)	23.0 (17.0–30.4)^c^	26.6 (23.3–30.1)
**Has paid sick leave**	22.6 (17.4–28.8)	30.4 (27.6–33.3)	16.9 (11.4–24.3)^c^	27.3 (24.0–30.9)
**Effect of sun exposure for 1 hour while unprotected**				
Severe/moderate sunburn	29.9 (23.3–37.3)	26.4 (23.3–29.7)	32.1 (24.6–40.7)	25.9 (22.7–29.4)
Mild sunburn	23.8 (18.4–30.3)	22.7 (20.0–25.7)	17.1 (11.7–24.4)	22.4 (19.3–25.8)
Turn darker without sunburn	28.4 (22.4–35.3)	33.5 (30.1–37.0)	33.8 (25.9–42.7)	34.2 (30.5–38.1)
Nothing would happen to my skin	17.9 (13.2–23.8)	17.5 (14.8–20.5)	17.0 (10.8–25.6)^c^	17.5 (14.4–21.1)
**Effect of sun exposure every day for 2 weeks while unprotected**				
Burn repeatedly or freckle	11.2 (7.9–15.7)^c^	11.1 (9.0–13.6)	^—c^	9.5 (7.2–12.4)
Mild tan	36.3 (29.9–43.2)	31.1 (27.8–34.7)	34.5 (25.1–45.2)	31.3 (27.7–35.2)
Moderate tan	35.9 (28.2–44.5)	37.9 (34.6–41.4)	31.6 (22.0–43.0)	38.8 (34.9–42.8)
Very dark tan	16.6 (11.5–23.4)	19.9 (17.0–23.1)	21.9 (14.6–31.6)^c^	20.4 (17.0–24.3)
**Number of sunburns in past year**				
0	64.1 (56.4–71.1)	64.5 (61.0–67.9)	67.6 (57.2–76.5)	65.1 (61.4–68.6)
1	18.8 (13.9–25.0)	16.2 (13.6–19.1)	18.0 (11.3–27.6)^c^	17.7 (14.8–21.0)
≥2	17.1 (12.4–23.0)	19.3 (16.7–22.2)	14.4 (9.8–20.6)^c^	17.2 (14.6–20.2)

Abbreviations: CI, confidence interval; GED, general equivalency degree.

a Industry refers to employer type of business or work (eg, dairy farm), whereas occupation refers to type of work (eg, farm hand).

b Respondents with a personal history of skin cancer (melanoma, nonmelanoma, or other/don’t know) (n = 102), family history of melanoma (n = 38), or a response of “do not go out in the sun” for any question (n = 218) were excluded.

c Estimates with a sample size <30 are not reported; estimates with a sample size of 30–59 are indicated and should be interpreted cautiously.

d Associate degree from occupational, technical, vocational, or academic program.

e Northeast (Connecticut, Maine, Massachusetts, New Hampshire, New Jersey, New York, Pennsylvania, Rhode Island, Vermont); Midwest (Illinois, Indiana, Iowa, Kansas, Michigan, Minnesota, Missouri, Nebraska, North Dakota, Ohio, South Dakota, Wisconsin); South (Alabama, Arkansas, Delaware, District of Columbia, Florida, Georgia, Kentucky, Louisiana, Maryland, Mississippi, North Carolina, Oklahoma, South Carolina, Tennessee, Texas, Virginia, West Virginia); West (Alaska, Arizona, California, Colorado, Hawaii, Idaho, Montana, New Mexico, Oregon, Utah, Nevada, Washington, Wyoming).

f Medicaid, other public insurance, and other coverage among people younger than 65; dual eligible (Medicaid and Medicare), Medicare only, Medicare Advantage, and other coverage among persons aged ≥65.

Construction workers were more prevalent in the South, agricultural workers were more prevalent in the West, and the lowest overall prevalence of ACWs was in the Northeast. Although most of these workers had health insurance coverage, few had paid sick leave (by industry, 22.6% in agriculture and 30.4% in construction; by occupation, 16.9% in agriculture and 27.3% in construction). The distribution of sun-sensitivity factors was similar across groups. About half of workers in both groups reported that the effect of short-term unprotected sun exposure would be a mild, moderate, or severe sunburn. When asked about repeated unprotected sun exposure, most reported that the effect would be a moderate to very dark tan. About one-third had at least 1 sunburn in the past year. Workers in agricultural occupations had a lower prevalence of sunburn than other ACWs.

More than half of ACWs reported never using sunscreen when outside on a warm sunny day for more than 1 hour (Table 2 and Table 3). The prevalence of using sunscreen with an SPF of 15 or more always or most of the time was low and did not differ by industry (19.1% in agriculture and 19.6% in construction) or occupation (21.4% in agriculture and 15.1% in construction). Among workers who reported any sunscreen use, about one-third used an SPF of 45 or more most often, and the distribution of SPF use was similar across groups. The prevalence of seeking shade always or most of the time was slightly higher than the prevalence of sunscreen use but did not differ by industry (25.5% in agriculture and 25.6% in construction) or occupation (29.1% in agriculture and 24.5% in construction).

**Table 2:** Weighted Percentages (95% CI) of Sun-Protection Use Among Workers in Agricultural and Construction Industries, National Health Interview Survey, United States, 2015

When you go outside on a warm sunny day for more than 1 hour, how often do you . . .	Industry Group		*P *Value^a^
	Agriculture, Forestry, Fishing, and Hunting (n = 443)	Construction (n = 1,507)	
**Stay in the shade**			
Always/most of the time	25.5 (20.2–31.7)	25.6 (22.5–28.9)	.99
Sometimes/rarely	55.1 (48.9–61.1)	55.3 (51.8–58.8)	
Never	19.4 (15.1–24.6)	19.1 (16.3–22.3)	
**Wear a baseball cap or sun visor**			
Always/most of the time	54.8 (48.9–60.6)	46.1 (42.6–49.7)	.054
Sometimes/rarely	21.9 (16.5–28.4)	25.6 (22.5–29.0)	
Never	23.4 (18.2–29.5)	28.3 (25.3–31.4)	
**Wear a hat that shades your face, ears, and neck (ie, wear a wide-brimmed hat)**			
Always/most of the time	25.9 (19.5–33.5)	15.6 (13.2–18.5)	.047
Sometimes/rarely	22.5 (17.0–29.1)	24.2 (21.1–27.5)	
Never	51.6 (43.7–59.5)	60.2 (56.7–63.6)	
**Wear a long-sleeved shirt**			
Always/most of the time	33.5 (27.2–40.5)	14.6 (12.3–17.3)	<.001
Sometimes/rarely	25.6 (20.4–31.6)	30.9 (27.8–34.1)	
Never	40.9 (34.8–47.3)	54.5 (51.2–57.8)	
**Wear long pants or other clothing that reaches your ankles**			
Always/most of the time	63.5 (56.2–70.2)	44.2 (40.6–47.9)	<.001
Sometimes/rarely	18.0 (13.4–23.9)	28.0 (25.0–31.3)	
Never	18.5 (13.8–24.3)	27.7 (24.4–31.4)	
**Use sunscreen with an SPF ≥15** b			
Always/most of the time	19.1 (13.7–26.0)	19.6 (16.9–22.5)	.66
Sometimes/rarely	27.3 (20.9–34.7)	30.3 (27.3–33.5)	
Never	53.6 (46.4–60.7)	50.2 (46.6–53.7)	
**SPF of sunscreen used most often** b			
15–44	63.7 (52.2–73.9)	64.1 (58.7–69.2)	.95
≥45	36.3 (26.1–47.8)^c^	35.9 (30.8–41.3)	
**Regularly use sun protection** d			
Yes	80.8 (75.3–85.3)	66.6 (63.3–69.7)	<.001
No	19.2 (14.7–24.7)	33.4 (30.3–36.7)	
**Regularly use ≥1 type of recommended protective clothing** e			
Yes	70.9 (64.4–76.6)	50.7 (47.0–54.5)	<.001
No	29.2 (23.4–35.7)	49.3 (45.6–53.0)	
**Sun-protection behavior score** f			
3–4	9.3 (5.8–14.5)^c^	4.9 (3.5–6.9)	<.001
2–2.9	31.2 (26.5–36.3)	18.4 (16.0–21.1)	
1–1.9	43.2 (36.7–49.9)	48.0 (44.5–51.6)	
0–0.9	16.4 (12.3–21.5)	28.7 (25.7–31.8)	

Abbreviations: CI, confidence interval; SPF, sun protection factor.

a Determined by χ^2^ test.

b Respondents who reported using sunscreen with SPF <15 were excluded.

c Estimates with a sample size of 30–59 should be interpreted cautiously.

d Sun protection was defined as doing 1 or more of the following always or most of the time: staying in the shade, wearing a wide-brimmed hat, long-sleeved shirt, or long clothing to the ankles, and using sunscreen of SPF ≥15.

e Protective clothing was defined as wearing 1 or more of the following always or most of the time: wide-brimmed hat, long-sleeved shirt, or long clothing to the ankles.

f Range 0–4; average of scores from the following behaviors with 4 indicating always and 0 indicating never for all behaviors: staying in the shade; wearing a wide-brimmed hat, long-sleeved shirt, or long clothing to the ankles; and using sunscreen of SPF ≥15.

**Table 3:** Weighted Percentages (95% CI) of Sun-Protection Use Among Workers in Agricultural and Construction Occupations, National Health Interview Survey, United States, 2015

When you go outside on a warm sunny day for more than 1 hour, how often do you . . .	Occupation		*P *Value^ a^
	Farming, Fishing, and Forestry (n = 250)	Construction (n = 1,208)	
**Stay in the shade**			
Always/most of the time	29.1 (21.3–38.4)	24.5 (21.0–28.2)	.62
Sometimes/rarely	53.5 (44.5–62.3)	57.1 (53.0–61.1)	
Never	17.4 (11.5–25.4)^c^	18.4 (15.4–21.8)	
**Wear a baseball cap or sun visor**			
Always/most of the time	50.6 (41.3–59.9)	47.5 (43.6–51.4)	.53
Sometimes/rarely	24.9 (18.7–32.5)^c^	23.9 (20.5–27.6)	
Never	24.5 (18.3–31.8)	28.7 (25.3–32.2)	
**Wear a hat that shades your face, ears, and neck (ie, wear a wide-brimmed hat)**			
Always/most of the time	28.8 (19.7–40.0)	14.6 (12.1–17.6)	.04
Sometimes/rarely	18.3 (12.9–25.3)^c^	23.9 (20.6–27.5)	
Never	52.9 (42.8–62.9)	61.5 (57.4–65.5)	
**Wear a long-sleeved shirt**			
Always/most of the time	42.8 (34.4–51.7)	15.6 (12.9–18.7)	<.001
Sometimes/rarely	22.0 (15.5–30.2)^c^	29.5 (26.1–33.1)	
Never	35.2 (27.4–43.9)	55.0 (51.2–58.7)	
**Wear long pants or other clothing that reaches your ankles**			
Always/most of the time	64.6 (55.8–72.5)	47.5 (43.2–51.9)	.002
Sometimes/rarely	19.2 (13.5–26.6)^c^	24.9 (21.6–28.5)	
Never	16.2 (11.4–22.6)^c^	27.6 (23.9–31.6)	
**Use sunscreen with an SPF ≥15** b			
Always/most of the time	21.4 (14.4–30.6)^c^	15.1 (12.3–18.3)	.37
Sometimes/rarely	24.9 (17.3–34.4)^c^	29.3 (25.7–33.1)	
Never	53.8 (45.1–62.2)	55.6 (51.5–59.7)	
**SPF of sunscreen used most often** b			
15–44	69.6 (52.8–82.4)^c^	64.1 (57.1–70.6)	.52
≥45	30.4 (17.6–47.2)^c^	35.9 (29.4–42.9)	
**Regularly use sun protection^d^**			
Yes	80.0 (74.3–84.8)	66.3 (62.4–70.0)	<.001
No	20.0 (15.3–25.7)	33.7 (30.0–37.6)	
**Regularly use ≥1 type of recommended protective clothing^e^**			
Yes	70.5 (63.2–76.9)	53.0 (48.8–57.2)	<.001
No	29.5 (23.1–36.9)	47.0 (42.8–51.2)	
**Sun-protection behavior score** f			
3.0–4.0	12.2 (7.2–20.0)^c^	5.1 (3.4–7.4)^c^	.001
2.0–2.9	33.0 (26.3–40.6)	17.6 (14.8–20.9)	
1.0–1.9	37.0 (28.5–46.4)	47.7 (43.8–51.6)	
0–0.9	17.8 (12.7–24.3)^c^	29.6 (26.0–33.6)	

Abbreviations: CI, confidence interval; SPF, sun protection factor.

a Determined by χ^2^ test.

b Respondents who reported using sunscreen with SPF <15 were excluded.

c Estimates with a sample size 30–59 should be interpreted cautiously.

d Sun protection was defined as doing 1 or more of the following always or most of the time: staying in the shade, wearing a wide-brimmed hat, long-sleeved shirt, or long clothing to the ankles, and using sunscreen of SPF ≥15.

e Protective clothing was defined as wearing 1 or more of the following always or most of the time: wide-brimmed hat, long-sleeved shirt, or long clothing to the ankles.

f Range 0–4; average of scores from the following behaviors with 4 indicating always and 0 indicating never for all behaviors: staying in the shade; wearing a wide-brimmed hat, long sleeved shirt, or long clothing to the ankles; and using sunscreen of SPF ≥15.

The prevalence of regularly using at least 1 type of recommended protective clothing was significantly higher among agricultural workers by industry (70.9% in agriculture and 50.7% in construction [*P* < .001]) and occupation (70.5% in agriculture and 53.0% in construction [*P* < .001]) (Table 2 and Table 3). When protective clothing items were assessed individually, agricultural workers had a higher prevalence than construction workers of regular use across all items by industry and occupation. However, use of clothing items varied within groups. The most prevalent protective clothing behavior among ACWs was wearing long pants or other clothing that reaches the ankles. The regular use of pants (63.5%, 64.6%) among agricultural workers was about twice their regular use of a wide-brimmed hat (25.9%, 28.8%) or long-sleeved shirt (34%, 43%) by industry and occupation, respectively. Construction workers’ regular use of pants (44.2%, 47.5%) was about 3 times their use of a wide-brimmed hat (15.6%, 14.6%) or long-sleeved shirt (14.6%, 15.6%) by industry and occupation, respectively. By industry and occupation, almost twice as many agricultural workers reported wearing a baseball cap or sun visor as those who used a wide-brimmed hat; the difference was about 3-fold among construction workers. However, use of a cap or visor did not significantly differ between agricultural workers and construction workers by industry or occupation.

Although most agricultural and construction workers reported regularly using at least 1 recommended method of sun protection (by industry, 80.8% in agriculture and 66.6% in construction [*P* < .001]; by occupation, 80.0%, agriculture and 66.3% in construction [*P* < .001]), prevalence of regular use of multiple methods was low, especially among construction workers (Table 2 and Table 3). Workers in agricultural occupations were more than twice as likely as workers in construction occupations to have an overall sun-protection behavior score ranging from 3.0 to 4.0 (*P* = .001). However, average behavior scores were low across all groups (by industry, 1.7 in agriculture and 1.4 in construction; by occupation, 1.8 in agriculture and 1.4 in construction).

## Discussion

Agriculture workers had a higher prevalence than construction workers of almost all sun-protection behaviors by both industry and occupation. Prevalence of regularly seeking shade was similar across all groups (about 25%), which was lower than the national estimate of regular shade use of 37% (15). Although regular sunscreen use did not differ among groups by industry or occupation, all groups had a lower prevalence of use compared with the national estimate of 32% (15). All groups reported a higher prevalence of regular use of protective clothing compared with national estimates of use of wide-brimmed hats (14%), long-sleeved shirts (12%), and long pants or other clothing to ankles (28%) (15). The prevalence of protective clothing use among workers in agricultural occupations was more than twice as high for use of wide-brimmed hats (29%) and long clothing (65%) and 3 times as high for use of long-sleeved shirts (43%) compared with national estimates (15). 

Higher prevalence of protective clothing use may be due to injury prevention employer policies (eg, reducing chemical exposures). However, some policies, such as requirements for construction workers to wear hard hats, could be contributing to construction workers’ lower prevalence of wide-brimmed hat use compared with agricultural workers. Although wide-brim attachments for hard hats are commercially available, they are not widely used because they tend to reduce the worker’s vision of overhead hazards. Neck shades that can be worn or attached to the back of hard hats, caps, or visors may be a better alternative. Among all groups, ACWs were more likely to use caps or visors than wide-brimmed hats, and more than half reported using them. Sunburn was common and reported by about a third of workers studied, a prevalence similar to national estimates (15). Sunburn during adulthood significantly increases a person’s chances of developing melanoma (18). Although data on the anatomic sites (eg, neck, ears) of sunburn were not available for our study, melanomas can occur on parts of the body that are not protected by caps. A combined behavioral approach (eg, sunscreen, headwear) is important for adequate skin cancer prevention at these anatomic sites.

Results of our study indicate a need for sun-safety and skin cancer prevention efforts that target ACWs and their employers. Interventions that are highly effective at increasing sun-protection behaviors and decreasing sunburns among outdoor workers include educational, behavioral, and environmental approaches in addition to workplace policies that support sun-protection practices (13,19). For example, one study found that although exposure to an educational intervention did not increase construction workers’ sun-safety knowledge, it did significantly increase sun-safety behavior, such as increasing shade use when working outdoors (20). Although companies may include use of personal sun-protection practices in their institutional policies, few supply sun-protection equipment, and most existing policies do not explicitly state an intent to protect employees from excessive sun exposure (21). However, additional studies of local government organizations found that interventions that include personal contacts and theory-based training increased the likelihood of adoption of formal sun-protection policies and that adoption of sun-safe practices is not constrained by government budget or size (22,23). More research analyzing local, state, and national sun-safety policies is needed to understand their effects in both government and nongovernment organizations.

Although most skin cancer prevention programs and policy-driven activities to date have focused on children, adolescents, and young adults (eg, SUNucate, behavioral intervention policies in schools, minors’ access to indoor tanning) (24–27), opportunities exist to target older adults in high-risk groups. National Comprehensive Cancer Control Program awardees that have skin cancer prevention objectives can adapt their state plans to include targeted activities for outdoor workers when possible. For example, South Dakota’s Comprehensive Cancer Control Program has partnered with cities to implement a worksite UV protection model policy that helps outdoor workers understand the importance of limiting excessive UV exposure (28).

Employers can help protect outdoor workers from skin cancer by increasing awareness of sun-exposure risks, such as time spent outdoors during midday hours and when the UV index is high (29). Employers can also encourage employees to use multiple layers of protection against the sun (ie, wide-brimmed hats, long-sleeved shirt, long pants, sunscreen, sunglasses, and shade) while acknowledging that barriers exist to practicing sun protection. For example, men aged 18 to 44 have reported barriers to using sunscreen (eg, too greasy, laborious to reapply) (30). Allergies to chemical sunscreens may also be a barrier to use.

In addition to implementing educational interventions, employers can provide sun-protection resources (eg, protective clothing, headwear, sunscreen). Additional strategies include scheduling breaks in the shade; encouraging workers to reapply sunscreen throughout their shifts; providing shade tents, shelters, and/or cooling stations; decreasing UV reflection by covering bright or shiny surfaces; and creating work schedules that minimize sun exposure. Sample sun-safe workplace policy templates and other materials for employers are available and can help guide evidence-based worksite initiatives to promote employee sun safety (28,29). Many employers of ACWs are small businesses and may need assistance from health organizations in developing educational or administrative interventions.

Employers can also collaborate with occupational safety and health organizations to adapt or modify existing workplace wellness policies and training to include sun-safety information, such as employee programs focused on avoiding heat illness, because many sun-safety practices also help to prevent heat-related illnesses. Additionally, trainings can teach workers how to recognize the signs and symptoms of overexposure to UV radiation, and employers can encourage them to be role models for positive routine behavioral changes in their occupational, community, and family systems (29). One study found that Hispanic men aged 18 to 44 reported being more likely to talk to their family members and peers about skin cancer risk and prevention when they worked in outdoor jobs where employers encouraged use of sunscreen or protective clothing (30).

Our study had limitations. Our data were based on self-reported information, which yields several limitations, such as being subject to interviewer or reporting bias. Results should be interpreted with caution, because stratifying by agricultural and construction industries and occupations yielded small sample sizes for some categories. Inferences about causality cannot be made because of the cross-sectional survey design. Given limitations and small sample sizes in some subcategories of industries and occupations, we could not assess differences in detailed recodes. Therefore, results may not be generalizable to all ACWs, particularly those with exclusion characteristics (eg, skin cancer history). In addition, sun-protection behavior questions did not explicitly ask whether behaviors were practiced during leisure and/or occupational sun exposure.

Prevalence of use of sunscreen and shade was low, but prevalence of use of protective clothing was high among study participants. Findings may be used to improve occupational health approaches to skin cancer risk reduction among ACWs and to inform education and prevention initiatives that address skin cancer. Employers can help reduce occupational health inequities and protect workers by creating workplaces that promote sun-safe policies, provide access to resources that facilitate sun protection, and foster workplace cultural sun-safety expectations. Future research is needed to assess differences and links between occupational and recreational UV exposure, sun-protection behaviors, and related skin cancer risk among high-risk (eg, non-Hispanic white men) and minority (eg, women, blacks) ACWs.
